# Fiscal policies and regulations for healthy diets in Sri Lanka: an analysis of the political economy of taxation and traffic light labelling for sugar-sweetened beverages

**DOI:** 10.1080/16549716.2023.2280339

**Published:** 2023-11-29

**Authors:** Sunimalee Madurawala, Kimuthu Kiringoda, Anne Marie Thow, Nisha Arunatilake

**Affiliations:** a Health, Education & Labour Department, Institute of Policy Studies of Sri Lanka, Colombo, Sri Lanka; b Menzies Centre for Health Policy and Economics, The University of Sydney, Sydney, Australia

**Keywords:** Public health, NCD prevention, nutrition, SSB tax, TLL system

## Abstract

**Background:**

Unhealthy dietary patterns significantly contribute to rising non-communicable diseases (NCDs) in Sri Lanka. The government has implemented policy measures to promote healthy dietary patterns, including the traffic light labelling (TLL) system for sugar-sweetened beverages (SSBs) in 2016 and taxation on SSBs in 2017.

**Objectives:**

To analyse how ideas, institutions, and power dynamics influence the formulation and implementation of these two interventions, and to identify strategies for public health actors to advocate for more effective food environment policies in Sri Lanka.

**Methods:**

This study drew on Kingdon’s theory of agenda-setting and Campbell’s institutionalist approach to develop the theoretical framework. We examined the political economy at the policy development and implementation stages, adopting a deductive framework approach for data collection and analysis. Data were collected from documents and key informants.

**Results:**

NCDs and nutrition are recognised and framed as important policy issues in health-sector policy documents, and the SSB tax and TLL system are seen as means of improving diets and health. Sri Lanka’s commitment to addressing NCDs and nutrition-related issues is evident through these policies. The Ministry of Health led policy development, and key stakeholders were involved. However, there are opportunities to learn and strengthen policy in Sri Lanka and elsewhere. Limited involvement and commitment of some stakeholders in developing national policies, industry interferences, and other gaps resulted in weaker policy design. Gender considerations were also given minimal attention in policy formulation and implementation.

**Conclusions:**

To enhance the effectiveness of the policies and regulations to promote healthy diets in Sri Lanka, comprehensive policy coverage, multistakeholder involvement and commitment to national policies, balanced power dynamics, technical feasibility, government commitment backed with high-level political support, awareness, and knowledge creation, managing industry interferences, integrating gender considerations are crucial factors.

## Introduction

Governments play a crucial role in shaping people’s diets through policy action [1]. However, despite the expectation that evidence-informed nutritional policies will be adopted to reduce the burden of diet-related non-communicable diseases (NCDs) and improve the food environment, many government policies still reflect traditional viewpoints on agricultural production, industry support, food security, economics, and trade [2]. These traditional viewpoints often tend to prioritise economic and production-related aspects, sometimes at the expense of other considerations like health, environmental sustainability, or social equity. Strong government policies are crucial in achieving a healthy, profitable, equitable, and sustainable food system that benefits everyone. To have strong policies that translate evidence into action, governments must possess the necessary knowledge, capacity, commitment, and have good governance and partnerships in place to support such action [2].

The National NCD policy of Sri Lanka aims to mitigate the enduring health impacts of chronic NCDs by promoting healthy lifestyles, addressing risk factors, and providing integrated, evidence-based treatment options for diagnosed NCD patients [3]. The Ministry of Health (MOH) serves as the principal government agency tasked with addressing NCDs, ensuring policy coherence, and coordinating efforts among stakeholders [4,5]. In recent decades, NCD-related morbidity and mortality in Sri Lanka have surpassed that of communicable diseases [6]. In the year 2000, the estimated annual death rate per 100,000 people was 664.1 for NCDs and 101.3 for communicable diseases. By 2019, these figures had decreased to 508.9 for NCDs and 49.1 for communicable diseases, indicating a slower rate of decrease for NCD death rates compared to communicable disease death rates [7]. NCDs account for 83% of all deaths in Sri Lanka, posing significant health and economic consequences [8]. Despite having relatively modest health expenditures, Sri Lanka has made commendable progress in attaining a high standard of health [9]. However, the high prevalence of NCDs places immense pressure on the country’s health system and economy. NCD control and prevention accounted for 29.8% of total current expenditure on health in 2017, rising to 31.3% in 2018 [10]. Unhealthy dietary patterns are among the leading behavioural causes of escalating NCD incidences in Sri Lanka. For instance, 26.5% of school children aged 13–17 years consume carbonated soft drinks at least once daily [11]. The daily median free sugar intake is also alarmingly high among pre-school children, accounting for 21.1% of their total energy intake. This exceeds the World Health Organization’s (WHO) recommended limit for sugar intake, which advises keeping sugar intake to less than 5% of total energy intake [12].

In recent years, the government of Sri Lanka has implemented two globally recommended policy interventions to prevent diet-related NCDs: taxes and labelling (Box 1). For instance, the WHO identifies implementing front-of-pack labelling as a 'best buy' and taxing sugar-sweetened beverages (SSBs) as an effective intervention to reduce unhealthy diets [13]. Implementing these policies demonstrates the Sri Lankan government’s interest and commitment in addressing NCD and nutrition-related issues. Fiscal interventions (i.e. government policies or actions that involve the use of financial measures, such as taxation, subsidies, or budgetary allocations, to achieve specific economic or social objectives) are important in creating incentives to reduce dietary risk factors for NCDs and generate revenues for the government [14]. For instance, fruit and vegetable subsidies in low-income settings have been associated with increased sales, while food taxes on targeted products, primarily nonessential energy-dense food, were associated with higher prices and reduced sales [15]. Taxing SSBs is a proven and cost-effective, policy measure. It has the potential to reduce health inequalities related to diet, reduce disease burden, generate domestic revenue, improve productivity, and enhance social welfare [16]. Given that the demand for SBBs is price sensitive, SSB tax increases can lead to improved health outcomes, as seen for tobacco and alcohol tax increases [17]. Interpretive front-of-pack nutritional labelling (FOPNL) initiatives positively impact nutritional content understanding, healthiness perception of products, selection of products with better nutritional quality, and purchase intention [18]. It is a cost-effective measure to help consumers understand the nutritional quality of foods and orient them towards healthier food choices at the point of purchase as interpretative FOPNL conveys nutritional information in symbols, colours, or words [19]. The traffic light labelling (TLL) system, a type of FOPNL, simplifies decision-making [20] and promotes positive behavioural change towards healthier and sustainable meal choices [21].

**Box 1.** Case Study Policies for PEA: TLL system for SSBs and SSB Tax.

The TLL system for SSBs was introduced in Sri Lanka under Section 32 of the Food Act, No. 26 of 1980, in 2016. It classifies drinks based on their sugar content: red label for drinks that contain more than 11 grams (g) of sugar per 100 millilitres (ml), amber label for 2–11 g per 100 ml, and green label for less than 2 g per 100 ml. In 2019, the regulations were further changed, mandating the display of salt and fat levels along with sugar. In the same year, the TLL, the colour coding system was further expanded to solid and semi-solid food. The 2019 ministerial regulations stipulated that food that contains more than 22 g of sugar should display a red label. Those between 8-22 g of 100 g and below 8 g of 100 g should carry amber and green labels, respectively. However, the regulations exempted primary agricultural products, exported products and those sold in bulk.
In November 2017, a tax of 50 Sri Lankan cents per gram of sugar in SSBs was implemented to increase the price and discourage consumption. The tax led to a 30-50% price increase, and several companies reported reduced SSB sales [29]. However, lobbying and politics reduced the tax to thirty cents per gram of sugar (i.e. a 40% reduction) in 2018 [30]. There were further revisions to the tax rate afterwards. The current SSB tax rate is 12 Sri Lankan Rupees (LKR) per litre or thirty cents per gram of sugar (applies to drinks with more than 4 grams of sugar per 100 ml. The tax applies to carbonated soft drinks, energy drinks (sugar content is more than 6 g/100mlfrom 2020 October), and fruit or vegetable-based drinks (sugar content is more than 8 g/100 ml). The WHO, which provided technical support to Sri Lanka in developing the SSB tax policy, recommends adjusting the tax annually to account for inflation and real economic growth, as well as raising public awareness about the SSB tax [31].

Political economy embodies social, political, and cultural dynamics that shape power relations [22]. Policy development requires technical soundness and consideration of political factors [23]. Nutrition labelling and fiscal policy interventions are shaped by political economy dynamics that impact policy design and implementation. For instance, health oriented SSB taxation faced challenges from economic policy paradigms and industry resistance in sub-Saharan African countries [24]. Similarly, FOPNL regulations in Mexico faced opposition from transnational food and beverage companies [25].

Political economy analysis (PEA) helps understand a country’s context, as challenges in food and nutrition vary across nations. A PEA can identify effective policy responses for challenging development issues [26]. As many of the challenges confronted in implementing nutrition policies have stemmed from political economy sources, addressing these requires managing the political economy factors at global, national, and subnational levels [27]. For this, a deeper understanding of the broader political economy factors shaping the global nutrition landscape, and a systematic PEA that provides such understanding are required [27]. Learning from past experiences can inform future policy improvements and decisions in different contexts [28].

This study employed PEA to examine two recent policy interventions promoting healthy dietary practices in Sri Lanka (Box 1). The aim was to identify strategies for advocating effective food environment policy design and implementation locally and globally.

## Methods

### Study design and frameworks

The study employed a case study research methodology, commonly used in social sciences, to comprehend complex issues in real-life contexts [32]. We focused on the policy development and implementation stages of the selected two case study policies, as these have been in place for a reasonable time. To establish the theoretical framework, we adopted Kingdon’s theory of agenda-setting and Campbell’s institutionalist approach to PEA. Kingdon’s Theory focuses on problem conceptualisation, how policy solutions are proposed, and the role of politics and actors in policy decisions [33]. It particularly examines the problem identification (i.e. agenda-setting) stage of the public policy process, highlighting three converging streams: problem, policy, and political [34]. Campbell’s Institutionalist Approach emphasises understanding the ideas, paradigms, and stakeholder context that shape policy decisions [35]. Table 1 summarises the theoretical framework for our study.

**Table 1.** Theoretical framework.

	Development (agenda setting and policy design) Kingdon [33]	Implementation Campbell [35]
Ideas	Problem frames and policy frames (for the solution)	Responsibility for solution
Institutions	Political and sectoral policy responsibilities	Sectoral policy responsibilities
Power/influence	Stakeholders	Stakeholders
Evidence	Evidence pointing to opportunities for further improving governance structure and capacities.	Evidence for barriers in implementation

Source: Compiled by the authors based on Kingdon [33] and Campbell [35].

We followed the theoretical framework outlined in Table 1 to design the key informant interview (KII) guide. We also used this framework, for the subsequent analysis of ideas, institutions and actors, their interests, and power and influence of the actors during policy formulation and implementation stages of the case study policies.

Furthermore, we examined the recognition and framing of NCDs and nutrition in national policy documents. For this, we used the variables delineated by Thow et al. to assess the content of the selected policies [36]. These variables encompassed the following aspects: (I) articulated policy objectives, (II) pertinent content regarding the food environment, SSB taxation, and TLL system in SSBs (this encompassed various facets such as marketing, reformulation, healthy food production, consumption, processing/manufacturing of healthy food, retail of healthy food, public procurement of healthy food, healthy catering guidelines, trade policy, fiscal policy, linking agriculture to food provision, social marketing and health promotion campaigns, nutrition labelling, and nutrition education), (III) framing the problem and ‘appropriate solutions’, (IV) governance, specifically the sectors responsible and coordination mechanisms, (V) consideration of gender aspects, both in problem description and solution, and (VI) implementation details, including resource allocation, stakeholder identification, and monitoring and evaluation.

We also examined how key informants perceived the concept of gender and whether it had been integrated into the policy formulation process. Gender refers to the socially constructed attributes linked to women, men, girls, and boys. It encompasses the norms, behaviours, roles, and interpersonal connections associated with each gender category within a given society. It should be distinguished from sex, which pertains to the inherent biological and physiological attributes of females, males, and intersex individuals [37]. Gender is a crucial dimension that should be considered in food policy formulation and implementation [38]. AMT, SM, and NA developed the study design and theoretical framework.

### Data collection

We collected data from two sources: documents and key informants. For documents, we considered policy documents, including strategic plans and action plans, produced by government agencies (e.g. ministries, departments and authorities) after the year 2000. Our search for policy documents encompassed various sectors, including health and nutrition, agriculture, finance, trade, social welfare, education, urban planning, and development. We identified the policy documents through (1) online searches of relevant government agency websites; (2) suggestions provided by the key informants; and (3) direct requests made to government agencies.

For key informants, we considered five categories: government officials responsible for the policies (government), other government sectors related to policies or outcomes (government other), private sector actors (private sector), civil society organisations (CSO) actors and academia, (CSOs and academia), and relevant multilateral agencies (multilateral agencies). We selected participants purposively based on expertise and professional responsibilities and we also used snowball sampling. We reached out to the potential key informants formally through emails and letters introducing the researchers, the purpose of the interview and the research background. Upon confirmation, the KII guide was shared with key informants. In total, we conducted 26 interviews. The KIIs were conducted either in English or Sinhala based on the respondents’ preference. Sinhala is one of the national languages in Sri Lanka. We collected data through KIIs from August 2021 to February 2022. The KIIs were conducted either virtually using the Zoom platform or in-person, at key informants’ offices or homes, considering the constraints posed by the COVID-19 pandemic. No third party was involved in the interviews, and on average one interview lasted approximately an hour. The interviews conducted in Sinhala were translated into English and the subsequent analysis was done in English. While there were additional potential key informants in the government category, we decided to discontinue further interviews after observing that the information provided in the first five interviews was repetitive. The interviewers were also aware of biases but aimed to remain unbiased. All interviews were recorded with consent, transcribed, and supplemented with field notes.

### Analysis

Our study followed a deductive framework approach both for data collection and analysis. We chose this approach due to its suitability for focused research with specific questions and pre-defined issues [39]. When analysing data, we used thematic analysis. Thematic analysis is an analytical method used in qualitative research for identifying, analysing, and reporting patterns (themes) within data [40]. Accordingly, we derived four themes following our theoretical framework – ideas, institutions, power, and evidence. Each theme was analysed in relation to the policy development and implementation stages of the case study policies. For example, we analysed the ideas and how those ideas were manifested in problem framing and policy framing when developing the SSB tax policy. Furthermore, we applied triangulation to the data obtained from our two sources, documents, and key informants whenever it was possible to do so. For instance, we analysed both the documents and KII data to examine how the framing of NCDs and nutrition had been carried out at the policy development stage. SM and KK analysed the data gathered from the key informants.

To examine the recognition and framing of NCDs and nutrition as policy issues, we analysed the policy documents. We used a matrix spreadsheet in Microsoft Excel to organise information from policy documents.

## Results

We reviewed 16 national policy documents, including strategic and action plans to assess how NCDs, nutrition, and the case study policies were acknowledged and framed within those policy documents. Furthermore, we delved into the ideas, institutions, actors, their interests, and the dynamics of power among these actors throughout the policy formation and implementation phases of the case study policies. For this, we conducted 26 KIIs, categorised as follows: government (five), government-other (seven), private sector actors (four), CSOs and academia (seven), and multilateral agencies (three). Ten out of 12 key informants in the government and government-other categories held senior positions within their respective organisations during the interviews. Our key informants consisted of 15 males and 11 females.

### Policy framing and policy content

#### Recognition of NCDs and nutrition as policy issues

Our analysis of policy documents and stakeholder interviews revealed that NCDs and nutrition were well recognised and framed as important policy issues. Key informants acknowledged that unhealthy dietary patterns significantly contributed to the increasing incidence of NCDs in the country. The health sector policy documents extensively focused on NCDs and nutrition issues.

#### Attention on crucial aspects of public health policy

While most of the policy documents in the health sector recognised NCDs and nutrition as policy priorities, some critical elements of a public health policy were not adequately addressed in these documents. Funding sources, governance, gender sensitivity and stakeholder identification were not sufficiently mentioned. However, the reviewed policy documents did cover certain areas relatively well, such as healthy food consumption, production of healthy foods, social marketing and health promotion campaigns, nutrition labelling, nutrition education and retail of healthy food.

#### Recognition of financing measures

The review of policy documents found a lack of clarity and recognition in the policy documents regarding financing proposed strategies and actions. For instance, the National Health Strategic Master Plan 2016–2025 Preventive Services Programme – Food Safety states that the government will take monetary responsibility for improving food safety but does not specify the actual financial sources or mechanisms [41]. Similarly, many policy documents do not explicitly commit to adopting fiscal measures to achieve their targets.

#### Multisectoral involvement in framing broader national policies

Our analysis indicated limited recognition of NCDs and nutrition in national sectoral policies outside the health and agriculture sectors, such as trade, transport and urban development and planning. Even the national policies formulated by the Ministry of Finance and the Ministry of Trade, the key ministries relevant to our case study policies (i.e. Ministry of Finance for SSB tax and the Ministry of Trade for TLL system in SSBs) showed limited acknowledgement of NCDs and nutrition in framing their national policies. However, they did engage and participate when the MOH formulated policies relevant to them.

### Policy development

#### Ideas

The policy environment in the country was conducive to taking policy measures against NCDs when introducing both these policies. NCDs and nutrition were recognised as policy priorities, and a high-level political commitment was demonstrated to address these issues. Both the case study policies aimed to discourage SSB consumption, with taxation focused on reducing affordability and the TLL system promoting healthy choices at the point of purchase. Revenue generation was a secondary consideration for SSB taxation, and the tax was not earmarked.

#### Institutions, power, and influence

The MOH played a crucial role in instrumentalising the SSB tax policy and TLL system on SSBs. Several departments and units within MOH were actively involved in policy formulation processes.

Other stakeholders, such as the Department of Fiscal Policy and the Department of National Budget of the Ministry of Finance, the Consumer Affairs Authority, the Ministry of Law, the Sri Lanka Standard Institute, and CSOs, contributed to the SSB taxation policy. A similar collaboration occurred for the TLL system, with the Consumer Affairs Authority, Medical Research Institute, and the Industrial Training Institute. While broad national NCD and nutrition policies lacked multisectoral involvement, specific stakeholders were actively engaged in formulating these two policies. This is primarily because certain policies required specific stakeholders’ involvement, rather than NCDs and nutrition issues being well integrated into their institutional roles. For instance, the participation of the Ministry of Finance was essential for formulating the taxation policy.

The WHO provided crucial support to the MOH in policy development, offering technical inputs, training, and consultations for both policies. For the TLL system in SSBs, other multilateral agencies such as the Food and Agriculture Organization (FAO) and World Food Programme (WFP) also contributed.

High-level political commitment, demonstrated by the then Minister of Health, drove policy change, underscoring its importance.

… WHO supported the formulation of this policy. SSB tax in Sri Lanka is initiated by the WHO offering evidence-based support to countries. On the part of the government, the MOH and the Environmental and Occupational Unit of the MOH took the main responsibility. Industry consultations may have occurred at various stages, but the MOH made major decisions. The Minister of Health at the time was heavily involved … [Key informant from the government category]

Industry participation was visible in developing both policies, but with communication gaps, particularly in the case of the SSB tax policy. Some fruit-based manufacturers felt excluded from initial consultations.

Our analysis revealed significant industry influence during the development of these policies, particularly in the SSB tax’s development. Intense lobbying led to multiple revisions to the tax policy. The SSB tax introduced in 2017 at a rate of LKR 12 per litre or 50 cents per gram of sugar in the product, has undergone four revisions. The most recent revision to the policy occurred in October 2020. The current SSB tax rate is LKR 12 per litre or 30 cents per gram of sugar for beverages with more than 4 g per 100 ml.

Other stakeholders believed industry interferences affected the SSB tax policy’s effectiveness and deterred the policy’s expected outcomes, yet some noted the importance of having the tax even at a low rate to positively shape consumer behaviour.

During the TLL system development, industry concerns arose about sugar levels, labelling, and implementation costs. They requested a grace period, which was given and extended multiple times. Fruit-based beverage manufacturers pushed for a WHO-approved label, opposing a local design. They further highlighted that using the same label for carbonated and fruit-based beverages is unfair as fruit-based beverages have natural sugar. Concerns had been raised about the potential for distortion in the regulation by carbonated beverage manufacturers.

Despite the industry influence, the then Minister of Health supported these policies. Interviewees mentioned the Minister’s consistent support given to this issue. Interviewees suggested that another contributing factor to his stance was the support received from the then Executive President (2015–2019), who shared a similar vision regarding the consumption of sweetened beverages [42].

### Policy implementation

#### Ideas

Views on the acceptance of case study policies, understanding, and the impact varied. Some noted positive public reception, while others highlighted minimal public awareness. There is a lack of public understanding of the SSB tax’s intent, with many believing it aimed to increase government revenue. Furthermore, the tax amount was considered inadequate to significantly alter consumer buying patterns, as expected.

*…* The tax that was imposed was exceedingly small, especially compared to other countries; it was very minimal. It was not sufficient to make a significant impact on the buying patterns of people*…* . [Key informant from the multilateral agency category]

The key informants emphasised the need to complement this fiscal policy measure with other interventions, such as rigorous public awareness campaigns and promoting behavioural changes.

#### Perceived impacts

The industry responded to these policies in various ways. For the SSB tax policy, the industry lobbied for tax reductions, reformulated products, and engaged in heavy advertising. The tax led to a drop in sales, prompting companies for product reformulations and introducing healthier options.

For the TLL policy, its impact on consumer behaviour was perceived as minimal by most key informants including the industry. Lack of awareness of this intervention, lack of health consciousness of the public in general, insufficient attention to the information given on labels, and intensive advertising by the industry were mentioned as reasons for the limited effect.

*…* Only a few concerned people make purchases based on the TLL. Others will continue to purchase the brands they are already used to. The propaganda of the industry is compelling*…* . [Key informant from the government category]

The industry responded to the TLL regulation by reformulating and introducing new products. According to the key informants representing the industry, the impact of the TLL system was less compared to the SSB tax.

*…* I don’t think that the industry was affected like how we were affected by the SSB tax. The industry adjusted itself to the TLL system. They changed their recipes, and it worked out *…* . [Key informant from the industry category]

Industry key informants emphasised the potential economic repercussions of decreased sales of fruit-based beverages resulting from these policies. They argued that implementing policies like the SSB tax and TLL system could pose sustainability challenges for the industry, potentially jeopardising the livelihoods of thousands of individuals involved in the fruit-based beverage supply chain in Sri Lanka.

#### Institutions, power, and influence

The SSB tax policy is enforced by the Minister of Finance, implemented by the Department of Customs (DOC), and guided by the MOH. The DOC handles tax collection imposes fines for tax evasion and manages other legal matters. Manufacturing companies are required to register with the DOC and submit quarterly reports.

The Consumer Affairs Authority leads the implementation of TLL system, which is monitored by the public health inspectors. Respondents acknowledged that the TLL system requires a collective effort, with all stakeholders adhering to the guidelines to ensure effective implementation. Consultations with key informants did not reveal any indications of interferences during the implementation phase.

Barriers to enhance the impact of the SSB tax policy in Sri Lanka include industry interferences, lack of nutrition concerns by the public and government, intense advertising by the industry, coordination challenges, and limited laboratory facilities.

*…* The companies run strong advertising campaigns, which surpass the government’s efforts. They also sponsor events, and the influence of such sponsorship and advertisements can challenge policy implementation *…* [Key informant from CSO and academia category]

Another crucial finding from the analysis is the absence of an earmarking mechanism for SSB tax revenue in Sri Lanka. Surprisingly, none of the respondents possessed knowledge regarding the revenue generated from the SSB tax. Strengthening the SSB tax policy includes obtaining legal support during drafting, enhancing stakeholder engagement, and utilising tax revenue for regulations, raising awareness, and promoting behavioural changes. Creating public awareness about sugar’s health hazards and the tax’s purpose is vital for improving consumer response, opined the stakeholders.

Implementing the TLL system for SSBs faced challenges such as technical gaps in the regulation (e.g. broad amber colour range, potential use of artificial ingredients to lower sugar levels, confusion with fat and salt levels) and industry practicalities (e.g. limited lab facilities and label printing costs). Moreover, lack of consumer awareness and industry interference are significant factors undermining the regulation’s effectiveness.

#### Integrating gender dimension into policy development and implementation

Depicting the same deficiency in recognition of gender dimension in the policy documents, only a limited number of respondents (three with two from multilateral agencies) recognised the importance of integrating gender into policy development and implementation. These respondents highlighted gender disparities in disease prevalence, nutritional requirements, and food consumption due to biological and cultural influences. They emphasised the necessity for gender-sensitive health and nutrition policies and enhancing stakeholder awareness. Majority of the respondents lacked a comprehensive understanding of the gender dimension and its significance within NCDs, nutrition, and food environment policies. They often equated gender solely regarding biological differences between sexes and variations in nutritional needs. While some respondents expressed satisfaction with the presence of women in policy discussions, doubts persisted about whether these women’s voices and concerns received sufficient attention or were adequately addressed during these discussions.

*…* In technical bodies, there is little understanding of gender. For example, the effect of gender on food choices is not considered. The understanding lacks how gender differences affect life at a practical level. Some policies are very theoretical, and the practicality of those policies is overlooked. This has led to people losing faith in policies *…* [Key informant from multilateral agency category]

## Discussion

### Summary of key findings

Through the analysis of national policy documents pertaining to NCDs and nutrition, we discovered that these issues were prominently acknowledged and framed, especially in documents produced by the health sector. However, it became evident that some critical elements, such as funding sources, financing mechanisms, governance, gender sensitivity, and stakeholder identification, received inadequate attention. Data obtained from key informants shed light on the policy development and implementation phases of the case study policies. In policy development. The MOH played a pivotal role and stakeholders actively engaged. However, this dynamic engagement was not observed in the formulation of broader national policies, and industry representation in exhibited communication gaps. High-level political commitment was notable during this phase. The impact of these policies on consumer behaviour patterns at the implementation stage received mixed feedback. The industry’s response differed for the two policies, with the TLL regulation having a lesser impact than SSB taxation. Implementation challenges for the SSB taxation policy were primarily linked to industry interference and a lack of awareness among the public and stakeholders. Conversely, the implementation hurdles for TLL regulation were mostly related to technical issues and resource constraints. The analysis found a lack of attention to gender considerations at all levels, policy framing, developing, and implementing.

### Interpretations and comparison to existing literature

Leadership efforts by the health sector in Sri Lanka have resulted in implementing two exemplary policies focusing on NCDs, nutrition, and the food environment: taxation and TLL system in SSBs. Stakeholder engagement during the process of developing these two policies was comparatively higher than that in developing broader national policies on NCDs and nutrition. Global experience suggests that cross-sectoral engagement is critical, particularly in adopting the SSB tax [43]. NCDs and nutrition are currently perceived as health-specific matters, with other sectors and ministries not fully integrating them into their policy formulation. The active involvement observed in the case study policies stemmed from their specific nature, facilitating the identification of relevant stakeholders and their designated responsibilities. For instance, the Ministry of Finance’s involvement in the SSB tax policy and the Industrial Training Institute and Consumer Affairs Authority’s participation in the TLL system for SSBs were due to their technical responsibility for the policy instruments used. Nonetheless, this level of engagement does not translate to sustained multisectoral involvement in NCDs and nutrition or comprehensive integration of these issues into the agendas of respective ministries.

Our study also revealed limited civil society representation during the policy development stage. Civil society participation in the health policy process is important for many reasons, such as delivering services beyond governmental capacity, mediating problematic policies, innovative problem-solving, and advocacy for social change [44].

Both multisectoral engagement and multistakeholder engagement are crucial for achieving the desired outcomes of a policy. While multisectoral engagement comprises the setting up of a framework to enable policy coherence across relevant governmental sectors, multistakeholder engagement calls for interaction with other stakeholders (such as the private sector and civil society) when and as appropriate, under government stewardship to achieve national NCD objectives [45]. The absence of such engagement during policy development leads to conflicts during implementation, hampering effectiveness and wasting resources. These conflicts would deter the effective implementation of the policy and cause resource wastage. For robust multistakeholder engagement, it is vital to identify stakeholders, develop engagement strategies for each stakeholder category, and establish suitable governance structures [46]. Enhancing multisectoral engagement involves mechanisms like fostering whole-of-government action against NCDs, ensuring political commitment, integrating health concerns into relevant-sector activities, and reinforcing responsible stewardship [47]. Political commitment is crucial in implementing the SSB taxation policy in Sri Lanka. Generating political and system commitments requires sustained efforts from policy entrepreneurs and champions [48]. Effective nutrition actor networks, strong leadership, civil society mobilisation, supportive political administrations, societal change and focusing events, cohesive and resonant framing, robust data systems and available evidence are key factors that drive and sustain a commitment to nutrition [49]. Hence, it is vital to strengthen these supportive factors to maintain the government’s focus and commitment to nutrition.

The rationale behind government policies and interventions should be clearly communicated to the public. For example, our analysis found that the motivation for introducing SSB taxes has not been distinctly conveyed to the public yet. The people do not see it as an intervention to disincentivise SSB consumption but as an effort to increase government revenue. Moreover, such communication is crucial as it signals the government’s stance on the issue and indicates its priorities. Moreover, when the industry has intensive marketing campaigns, the government must have proactive and frequent communication with the public. Such actions can increase support for policy implementation and reduce the likelihood of public mistrust [50].

Investing in public awareness campaigns that explain the benefits of SSB taxes and provide information about how tax revenues will be used before and after a tax proposal is vital to gain the expected impact of the policy [51]. Currently, we could not observe such occurrence of awareness raising related to the two case study policies. Public support tends to increase when policymakers provide detailed information about the allocation of tax funds and communicate the potential benefits to the public [52]. Moreover, creating awareness across government sectors and among stakeholders is also imperative. In the case of SSB taxes, proactive public and policy engagement, being prepared to counter public opposition in the media, and forming an alliance in which multiple voices support SSB taxation and policy and institutional strategies are vital in countering industry opposition [53].

While industry representation at the policy development stage can be considered an essential measure in resolving conflicts and ambiguities, there are concerns regarding industry involvement in formulating public health policies. For instance, it has been pointed out that the industry should not have a seat at the policy table due to inherent and unavoidable conflicts of interest but should provide their input or raise their concerns at the public consultations during the policy development stage [54]. The decision to incorporate industry comments lies within the government’s discretion, aiming to ensure the enforceability of the tax and address any potential loopholes. It is recommended to have governance mechanisms to manage conflict of interest and to assist governments in managing industry influence sufficiently [54].

Our analysis revealed that the industry challenged the case study policies primarily due to the perceived economic impact, particularly concerning the potential loss of employment opportunities for individuals involved in the supply chain. The threat of reduced economic activity is a commonly raised argument by vested interests regarding SSB taxes, in this case as well as in other countries. Specifically, the industry argues that such tariffs could result in significant employment costs in beverage manufacturing, crop agriculture, and food retail sectors. They also claim that the overall economy may suffer, leading to reduced foreign investment [55]. For instance, during the public consultation process for introducing the SSB tax in South Africa, one of the core claims raised by the industry against the policy was the perceived economic harm. Their argument focused primarily on the potential negative impact of the SSB tax on employment and the country’s economic growth [56]. However, independent empirical evidence is scarce to substantiate these concerns. Independent studies have found no significant changes in employment rates following the implementation of SSB taxes. This is because job losses observed in the taxed sector are usually balanced by job gains in non-taxed sectors [57].

Factors such as a robust public health coalition with a unified position and strong media presence, technical preparedness, and increased lobbying transparency are potentially effective strategies for preventing or countering such kind of industry interference [58].

There have been recommendations for earmarking revenue generated from SSB taxes, which was not done in Sri Lanka but is a viable option to maximise health gains through the strategic use of income. Earmarking can be done in several ways, such as strengthening health promotion actions (e.g. funding education campaigns, providing healthy food subsidies) and limiting the regressive impact of taxation (when the effect is indeed regressive) [59]. Further, earmarking will enhance the transparency of the taxation process and use of revenues, increasing the acceptability of the tax by politicians and the public [59]. The earmarking of tax revenues generated from the SSB taxes is used in many countries. The general prevention fund in French Polynesia, drinking water in Mexico, and preschool children’s education in Philadelphia are some examples [60]. However, since earmarking has pros and cons and is highly context-specific [61], a careful and closer examination of the country’s health system’s political economy is needed before introducing such a measure for Sri Lanka.

Our study identified a lack of scientific evidence on consumer awareness and acceptance of nutrition and food policies and regulations, consumers’ consumption and behaviour patterns, and the effectiveness of the policies in the context of Sri Lanka. As such, it is crucial to encourage experts in the field, academia, and research institutes to create new knowledge on those aspects. Moreover, ensuring those research findings are well documented, disseminated, and used in policymaking is equally essential. Reich (2019) noted that in the global context, there is significant room for expansion in NCD-related academic theory, data collection, case study analysis, focused comparisons, and documentation of real-world practices [62]. Filling these knowledge gaps is vital to understand how effective NCD policies overcome corporate and bureaucratic resistance obstacles and how social movements successfully pursue reforms.

Our analysis reveals limited integration of gender dimensions in policy framing and policy development processes. Most respondents did not demonstrate a clear understanding of gender and did not recognise the importance of acknowledging gender differences and gender norms in policy formulation. Given that gender responsiveness would enhance the acceptance, relevance, and effectiveness of health promotion interventions [63], all the stakeholders should pay attention to increasing gender sensitivity in the policy process. Health promotion programmes operate within a specific gender system that shapes their implementation and impact while simultaneously having the capacity to impact the gender system itself, thereby contributing to a broader environment that promotes enhanced health and well-being [64]. Applying a gender lens at the onset of the policy process (i.e. at the agenda setting and policy designing) is essential to ensure gender consideration throughout the policymaking process. Adopting complexity-informed design, implementation, and evaluation strategies to create gender-transformative health promotion policies helps integrate gender dimensions into the NCDs and nutrition policies [64].

### Strengths and limitations

This study offers valuable insights into how the political economy influences the development and implementation of fiscal policies and regulations for healthy diets within the context of Sri Lanka. These insights are grounded in empirical data related to two selected case study policies. In a context where Sri Lanka grapples with significant challenges in terms of health, economics, and social implications due to the rising prevalence of NCDs, this study equips public health actors with strategies to advocate for more effective food environment policies. The research also highlights the opportunities available for Sri Lanka to further strengthen the development and implementation of food environment policies. Furthermore, this study contributes to the existing literature, particularly in a context where literature on the political economy related to sugary beverages in low- and middle-income countries is limited [62]. This study has two main limitations. One significant constraint was the limited representation from the industry, particularly from carbonated beverage manufacturers. As a result, the perspectives presented may be more oriented towards health concerns, with less engagement regarding the economic dimension. Additionally, due to documentation gaps in certain government institutes, we were unable to access a few relevant policy documents during the initial screening stage of the national policies. For instance, we could not obtain the latest National Agricultural Policy 2022, and this may have implications for our policy framing analysis. However, we obtained the majority of documents, which provided a strong reference point against which to understand the case study, and to triangulate the interview data.

## Conclusions

This study analysed the political economy of SSB taxation and TLL labelling systems in SSBs in Sri Lanka. NCDs and nutrition are well recognised and well framed as policy issues in the policies produced by the health sector. In this case, the health sector was able to lead on best-practice policy interventions with support from key actors. However, nutrition has not been integrated into the core objectives of other sectors, limiting the policy space for addressing NCDs and nutrition issues. Both the SSB taxation and TLL system in SSBs policies were developed through a consultative process, yet industry influence resulted in multiple revisions, which affected the effectiveness of these policies. Lack of consumer awareness, technical gaps in the policies and implementation difficulties have impeded the opportunity to make a meaningful impact through these policies on consumer behaviour of choosing unhealthy foods. Conceptual understanding of gender is considerably low among the stakeholders, and integrating gender into policy framing and policy development is minimal. Comprehensive policy coverage, involvement, and commitment of all the stakeholders, mutual trust between stakeholders, and balancing the power dynamics among them, technical integrity and feasibility of the policies, and the government’s commitment to take proactive steps in promoting healthy diets backed by high-level political support are critical factors which create an opportunity to strengthen the public health policies at the policy development stage. At the policy implementation level, creating awareness, managing industry interferences, and generating scientific evidence are crucial in maximising the benefits of fiscal policies and regulations to promote healthy diets. Integrating gender considerations when articulating and implementing would also increase the effectiveness of public health and nutrition policies.

## Paper context

We examined the political economy of taxation and traffic light labelling for sugar-sweetened beverages in Sri Lanka. Despite stakeholder involvement in these cases, their engagement and commitment were limited in broader national policy development. Industry’s interference and other policy gaps resulted in weaker policy designs. Gender considerations were also overlooked. For effective fiscal policies promoting healthy diets in Sri Lanka, ensuring comprehensive policy coverage and government commitment, involving stakeholders, maintaining technical integrity, addressing industry interference and considering gender considerations are crucial.
